# Does mandibular advancement ortho-surgical procedure cause condyle changes? A case-series analysis

**DOI:** 10.1590/2177-6709.29.3.e2423261.oar

**Published:** 2024-07-08

**Authors:** Alline Birra Nolasco FERNANDES, Luíza Trindade VILELA, Taísa Figueiredo CHAGAS, Antônio Carlos de Oliveira RUELLAS, Cláudia Trindade MATTOS, Margareth Maria Gomes de SOUZA

**Affiliations:** 1 Private Practice (Rio de Janeiro, Brazil).; 2 Federal University of Rio de Janeiro, Department of Pediatric Dentistry and Orthodontics (Rio de Janeiro, Brazil).; 3 Federal Fluminense University, Department of Pediatric Dentistry and Orthodontics (Rio de Janeiro, Brazil).

**Keywords:** Mandibular advancement, Condyles, Tomography, Avanço mandibular, Côndilos, Tomografia

## Abstract

**Introduction:**

Mandibular advancement surgery corrects bone bases while establishing patients’ functional and aesthetic rehabilitation. However, little is known about the results of this procedure in the structures that make up the stomatognathic system, as the condyles.

**Objective:**

This study aimed to evaluate the structural and positional changes of mandibular condyles in ortho-surgical patients who underwent mandibular advancement surgery.

**Material and Methods:**

A prospective investigation was conducted with cone-beam computed tomography images. Using Dolphin Imaging® software, seven ortho-surgical patients with Angle Class II malocclusion and mandibular deficiency were evaluated. The images assessed were obtained at pre-surgical phase and after, at least, 1 year of the procedure. To study the structural and positional changes of condyles, linear and angular measurements were obtained, and the right and left sides of patients were compared. Descriptive statistical analysis was performed and, in order to verify possible significant differences, normality tests (Kolmogorov-Smirnov) were applied, followed by a paired *t-*test to define significance.

**Results:**

For all measures evaluated in this study, no statistically significant differences were found.

**Conclusion:**

The ortho-surgical procedure performed did not change the structure and position of the condyles of patients who underwent surgical mandibular advancement. Right and left mandibular condyles behaved similarly, suggesting stability and condylar adaptation after surgery.

## RESUMO

**Introdução:**

A cirurgia de avanço mandibular corrige as bases ósseas e estabelece a reabilitação funcional e estética dos pacientes. Entretanto, pouco se sabe sobre os resultados desse procedimento nas estruturas que compõem o sistema estomatognático, como os côndilos.

**Objetivo:**

Este estudo teve como objetivo avaliar as alterações estruturais e posicionais dos côndilos mandibulares em pacientes ortodôntico-cirúrgicos submetidos à cirurgia de avanço mandibular.

**Material e Métodos:**

Foi realizada investigação prospectiva com imagens de tomografia computadorizada de feixe cônico. Utilizando o *software* Dolphin Imaging®, foram avaliados sete pacientes ortodôntico-cirúrgicos com má oclusão Classe II de Angle e deficiência mandibular. Foram avaliadas imagens da fase pré-cirúrgica e de, no mínimo, um ano após o procedimento. Para estudar as alterações estruturais e posicionais dos côndilos, foram obtidas medidas lineares e angulares e comparados os lados direito e esquerdo dos pacientes. Foi realizada análise estatística descritiva e, para verificar possíveis diferenças significativas, foram aplicados testes de normalidade (Kolmogorov-Smirnov), seguido de teste *t* pareado, para definição de significância.

**Resultados:**

Para nenhuma das medidas avaliadas nesse estudo foram encontradas diferenças estatisticamente significativas.

**Conclusão:**

O procedimento ortodôntico-cirúrgico realizado não alterou a estrutura e a posição dos côndilos dos pacientes submetidos à cirurgia de avanço mandibular. Os côndilos mandibulares direito e esquerdo comportaram-se de forma semelhante, sugerindo estabilidade e adaptação condilar após a cirurgia.

## INTRODUCTION

Orthodontic treatment aims to correct dental positions and achieve optimal occlusion, by creating harmony between dental elements and bone bases. Orthodontics alone is not sufficient to resolve severe skeletal malocclusion, which often requires orthognathic surgery for satisfactory correction of maxillary discrepancies.^1^ ^,^ ^2^

However, post-surgical stability is a source of concern for both oral and maxillofacial surgeons and for orthodontists. Post-operative occlusion and condylar position alteration are among the main causes of surgical recurrence.^3^ Control of condylar position is critical during and after surgery. Post-operative condylar position can be affected by multiple factors, such as the rotational movement of the distal segment of the mandible, the tension balance of surrounding muscles, the surgical technique, the method of fixation, and surgeon expertise.^4^

Cone-beam computed tomography (CBCT) is the best modality to evaluate bone and condylar position changes, as it allows for an evaluation of skeletal relationships that could not be evaluated in a two-dimensional way. This method has the potential of highlighting associations between structural change and surgical correction stability.^5^ ^,^ ^6^

Structural and positional changes of condyles and mandibular fossa in cases of mandibular advancement are not well covered in the literature. Systematic reviews demonstrate that comparing the results obtained in selected studies is quite challenging^7^ ^,^ ^8^ ^,^ ^9^, due to the numerous methodological flaws, including comparison of patients with different malocclusions and difficult evaluation through images.^7^ Therefore, the objective of this study was to evaluate, using CBCT, the existence of structural and positional changes, in addition to changes in dimensions of the mandibular condyle, and to observe if it underwent a process of physiological adaptation after mandibular advancement surgery, in a follow-up period of at least one year.

## MATERIAL AND METHODS

This study consists of a series of cases of patients suffering from the same type of malocclusion, who underwent mandibular advancement orthognathic surgery at the same hospital, conducted by the same professionals, and following the same technique.

### SAMPLE

This prospective study was approved by the Research Ethics Committee of the Instituto de Estudos em Saúde Coletiva (IESC) at Universidade Federal do Rio de Janeiro (UFRJ) under protocol number 0045.0.239.000.10. All patients signed an informed consent form.

Sample size calculation was based on standard deviation (SD=1.5mm) for linear measurements and standard deviation (SD=3.3°) for angular measurements, according to a previous study^2^. The calculation considered a minimum detectable difference of 2 mm in linear measurements and 4 degrees in angular measurements, α = 0.05 and β = 0.2. The case series consisted of 7 patients (6 women and 1 man).The sample was selected among patients who had mandibular advancement surgery planned to be performed at Pedro Ernesto University Hospital, linked to the Universidade do Estado do Rio de Janeiro (UERJ).

The inclusion criteria were: patients aged 18+, undergoing orthodontic treatment in pre-surgical phase, presenting Angle Class II malocclusion and mandibular deficiency (mean: SNB=74.3º). The treatment plan included mandibular advancement surgery with no anteroposterior movement of the maxilla Four patients underwent combined mandibular advancement surgery with genioplasty and maxillary replacement (counterclockwise rotation of the maxilla) and rigid fixation; the other three patients underwent isolated mandibular advancement orthognathic surgery with rigid fixation. Patients who had syndromes, previous craniofacial disorders, cleft lip and palate, any type of systemic involvement and/or patients with condylar malformation were excluded from the sample.

### IMAGES

The Dolphin Imaging^®^ software v. 11.5 (Dolphin Imaging, Chatsworth, California, USA) was used to read the DICOM tomographic files and to obtain a 3D image reconstruction and multiplanar reconstructions (MPR) in axial, coronal and sagittal views.

CT scans were requested at two opportunities: during the pre-surgical phase and at least one year after surgery. All CBCT scans were performed in the same radiological clinic (Radiologia Odontológica Doutor Murillo Torres, in Rio de Janeiro/RJ), using the same CT scanner (i-CAT 3D Dental Imaging System, Pennsylvania, USA), in order to obtain more standardized scans (120 kV, 5 mA, 13x17cm^2^ FOV, 0.4 mm voxel and 20s scan time).

For more standardized CT scans, the head orientation in all CBCT images followed the references in sagittal, axial and coronal planes, according to Weber et al.^28^

### MEASUREMENTS

**Figure 1:**
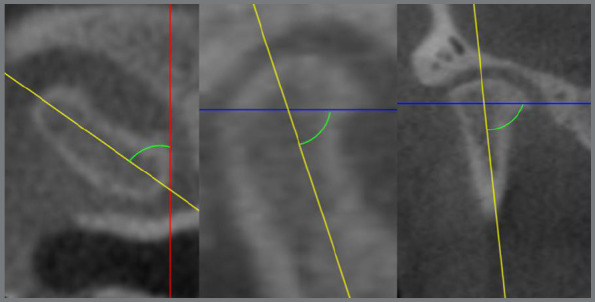
Angular measurements of the condyle in the axial, sagittal and coronal axes.

**Figure 2:**
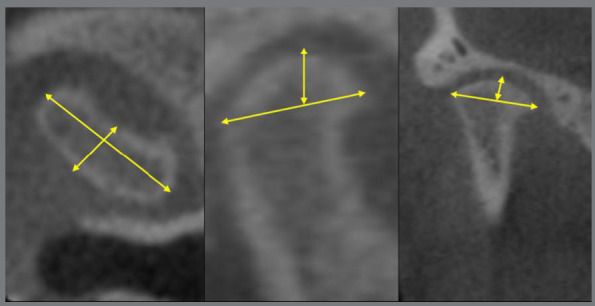
Linear measurements on the axial, sagittal and coronal axes.

Initially, sample characterization measurements of SNA and SNB were obtained, in order to prove both the anteroposterior mandibular alterations and the gonial angle. Subsequently, structure, position, and dimension of the condyle were measured (Table 1). The following angular measurements were performed: axial, sagittal and coronal condylar angles (Fig. 1). And the following linear measurements: condylar length, condylar depth; and axial, sagittal and coronal condylar height (Fig. 2).

**Table 1:** Description of angular and linear measurements of condylar structure, position and dimension.

Measurements	Measures evaluated	Abbreviations	Description
Angular	Axial condylar angle	Ax. ang.	The angle between the condylar axis and the midsagittal reference plane with the horizontal plane
	Sagittal condylar angle	Sag. ang.	The angle between the condylar long axis and FH in the sagittal plane (condylar long axis passing through the center of the condylar neck and the condyle center)
	Coronal condylar angle	Cor. ang.	The angle between the condylar long axis and FH in the coronal plane (condylar long axis passing through the branch to the center of the lateral pole of the condyle)
Linear	Axial condylar width	Ax. wid.	The greatest distance between the medial and lateral poles of the condyle in the axial plane
	Axial condylar depth	Ax. dep.	The greatest distance between the most anterior and posterior points of the condyle in the axial plane
	Sagittal condylar height	Sag. heig.	Vertical distance from the condyle-depth line to the most superior point of the condyle in the sagittal plane
	Sagittal condylar depth	Sag. dep.	The greatest distance between the most anterior and the most posterior points of the condyle in the sagittal plane
	Coronal condylar width	Cor. wid.	The greatest distance between the medial and lateral poles of the condyle in the coronal plane
	Coronal condylar height	Cor. heig.	Vertical distance from the condyle-width line to the most superior point of the condyle in the coronal plane

**Figure 3:**
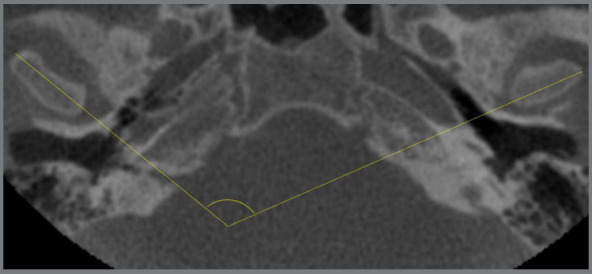
Measurement of the intercondylar angle between the longitudinal axes of the condyles.

**Figure 4:**
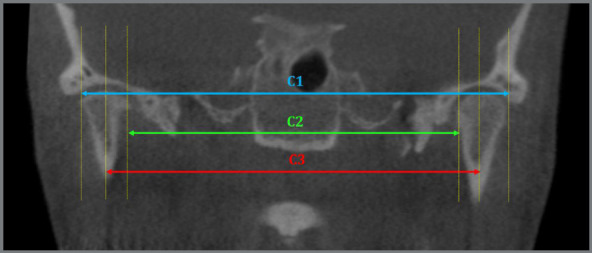
Linear measurement of intercondylar distance.

**Figure 5:**
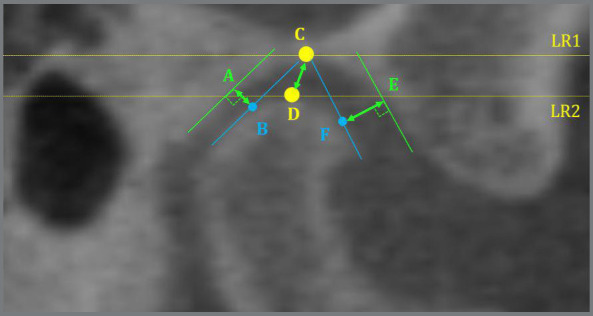
Linear measurements of condyle-fossa distance. Posterior: from point A (posterior superior point of the mandibular fossa) to point B (posterior superior point of the condyle head ). Superior: from point C (point of greatest concavity of the mandibular fossa) to point D (most anterior superior point of the condyle head). Anterior: from point E (most anterior superior point of the mandibular fossa) to point F (most anterior superior point of the condyle head).

Intercondylar measurements and the condyle-fossa distance were determined (Table 2). In the axial plane, the intercondylar angle between the longitudinal sides of the two condyles was measured (Fig 3). In the coronal plane, intercondylar measurements were evaluated through the distances between the latero-distal poles of the two condyles, between the latero-medial poles and the distance between the condylar centers (Fig 4). Distances between the condyle and the temporal fossa in sagittal plane were calculated, considering the posterior, superior and anterior regions (Fig 5).

**Table 2:** Description of intercondylar measurements and condyle-fossa distance.

	Abbreviations	Description
Intercondylar angle	IA	Crossing of the lines along the longitudinal axis of the condyle
Intercondylar distance	C1	Distance between lateral-distal edges
	C2	Distance between lateral-medial edges
	C3	Distance between condyle centers
Condyle-fossa distance	AR/AL	Distance between the mandibular condyle and the anterior part of the temporal fossa
	PR/PL	Posterior space between the fossa and the mandibular condyle
	MR/ML	Superior space between the fossa and the mandibular condyle

### STATISTICAL TREATMENT

For more reliable measurements and examiner calibration, 20% of the sample was randomly selected and the established method was repeated after a two-week interval. The Intraclass Correlation Coefficient (ICC) mean was 0.95. After data collection and tabulation, descriptive analysis (mean and standard deviation) was performed, and the Kolmogorov-Smirnov test revealed normal data. The paired *t*-test was applied to determine statistically significant differences (*p*<0.05) between the measures evaluated.

Structural and positional changes of mandibular condyles were evaluated by creating angular and linear measurements, as described in Table 1.

## RESULTS

When investigating changes in the condyle axis during descriptive analysis, the magnitude of the condylar axis was observed in the three planes of space. It should be noted that the values found were not statistically significant, as observed in Tables 3 and 4. Meanwhile, some measures showed slight tendency to alterations, such as R Ang Ax, R Ang Cor, L Ang Cor, L Wid Ax (Table 3) and PR (Table 4).

**Table 3:** Descriptive analysis of angular and linear measurements performed at T1 and T2, and comparative statistical analysis between T2 and T1.

Ang. meas.	T1	T2	T2-T1	p-value	ICC
R Ax Ang	70.40 (15.02)	70.13 (17.34)	0.27 (-6.72;7.27)	0.927	0.979
L Ax Ang	103.10 (11.94)	105.14 (87)	-2.04 (-5.55;1.46)	0.204	0.998
R Sag Ang	73.71 (7.11)	71.83 (4.68)	1.88 (-3.72;7.49)	0.442	0.947
L Sag Ang	74.27 (7.29)	70.80 (9.75)	3.47 (-1.16;8.10)	0.117	1
R Cor Ang	92.84(4.66)	89.10 (11.26)	3.74 (-8.36;15.85)	0.478	0.999
L Cor Ang	86.40 (3.48)	87.47(6.11)	-1.07 (-7.07;4.93)	0.678	1
SNA	81.99 (2.42)	82.11 (2.50)	-0.12 (-0.52;0.26)	0.456	0.989
SNB	73.81 (4.30)	78.04 (2.98)	-4.22 (-5.75;-2.70)	<.001	0.984
Linear meas.	T1	T2	T2-T1	p-value	ICC
R Sag Dep	15.66 (3.31)	15.23 (3.13)	0.42 (-0.45;1.30)	0.278	0.998
L Sag Dep	15.81 (2.81)	15.26 (3.07)	0.55 (-0.12;1.23)	0.091	0.751
R Sag Heig	8.70 (1.81)	8.73 (2.34)	-0.02 (-1.19;1.13)	0.954	0.976
L Sag Heig	8.53 (3.00)	7.50 (3.09)	1.02 (-0.51;2.57)	0.154	0.871
R Ax Dep	14.41 (2.13)	14.27 (2.34)	0.14 (-1.05;1.34)	0.781	0.998
L Ax Dep	15.07 (1.84)	14.64 (1.56)	0.42 (-0.21;1.06)	0.152	1
R Ax Wid	5.01 (1.45)	5.13 (1.50)	-0.11 (-0.89;0.66)	0.733	0.913
L Ax Wid	5.16 (1.22)	9.16 (10.76)	-4.00 (-13.89;5.89)	0.361	0.744
R Cor Wid	1034 (1.15)	10.09 (0.93)	0.25 (-0.46;0.98)	0.418	0.908
L Cor Wid	10.01 (2.02)	10.31 (1.98)	-0.30 (-1.13;0.53)	0.415	0.977
R Cor Heig	5.06 (1.35)	4.76 (0.86)	0.30 (-0.50;1.10)	0.399	0.941
L Cor Heig	4.56 (1.15)	4.74 (1.16)	-0.18 (-0.80;0.42)	0.487	0.991

**Table 4:** Descriptive analysis of intercondylar measurements and condyle-fossa distance measurements performed at T1 and T2, and comparative statistical analysis between T2 and T1.

	T1	T2	T2-T1	p-value	ICC
AR	2.39 (0.85)	2.36 (0.46)	0.02 (-0.46;0.52)	0.892	0.977
AL	1.64 (0.45)	1.94 (0.47)	-0.30 (-00.93;0.33)	0.288	0.927
PR	2.99 (1.57)	2.57 (9.96)	0.41 (-0.81;1.64)	0.441	0.936
PL	2.47 (1.29)	2.69 (0.95)	-0.21 (-0.75;0.32)	0.367	0.992
MR	2.56 (0.98)	2.36 (1.07)	0.20 (-0.40;0.80)	0.449	0.994
ML	1.97 (0.56)	2.07 (0.74)	-0.10 (-0.71;0.51)	0.704	0.975
IA	140.64 (17.92)	143.20 (17.91)	-2.55 (-11.97;6.85)	0.531	0.998
C1	110.63 (6.06)	109.19 (6.20)	1.44 (0.11;2.76)	0.037	0.982
C2	80.11 (3.30)	79.29 (3.33)	0.82 (0.35;2.01)	0.137	0.459
C3	94.79 (4.81)	93.50 (3.69)	1.28 (-1.37;3.94)	0.281	0.799
S	124.49 (6.16)	124.36 (5.35)	0.12 (-2.41;2.66)	0.905	0.9

When analyzing the statistical results obtained, it is possible to notice the absence of statistically significant differences, and descriptive analysis data showed that condylar configuration remained unchanged after surgery.

Measurements of right and left sides were also compared (Table 4), and the results obtained in the statistical analysis showed no significant differences between the variables at different times, suggesting that the mandibular condyles on both sides behaved similarly.

## DISCUSSION

When evaluating the results obtained in this study, it is possible to observe that the magnitude of the changes on mandibular condyles were not significant. A study by Catherine et al.^10^ corroborates our findings. Since these alterations are within the physiological limits, no clinical or radiological signs were found.^10^

The results obtained in terms of mandibular condyle rotation corroborate the findings of Carvalho et al.^11^ Small condylar rotations did not seem to have functional impairment, and apparently tend to decrease over time as a result of the adaptation process.^5^ ^,^ ^12^ ^-^ ^19^

In a systematic review, Barone et al.^20^ demonstrate that condylar resorption is a consequence of orthognathic surgery, but limited evidence is found. Additionally, significant condylar resorption may occur after orthognathic surgery of retrognathic mandible, regardless of the pre-surgical condition of the condyle.^21^ Meanwhile, supporting our findings, Barone et al.,^22^ in a study with Class III individuals undergoing ortho-surgical treatment with a 12-month follow-up, did not record any statistically or clinically significant condylar displacement.

The magnitude of mandibular condyle changes was not significant, although they tended to change in the right axial angle. The same tendency was observed by Hsu et al.,^23^ who affirmed that direction of mandibular surgery could contribute to different alterations of the condylar angle in the axial plane.

Surgeons may change the location of the mandibular condyle in the mandibular fossa during fixation.^24^ Rigid fixation has become a common procedure, and is one of the main causes of temporomandibular disorders.^24^ Post-operative condylar position is known to be affected by several factors, such as the rotational movement of the distal segment, the tension balance of surrounding muscles, the method of fixation, and surgeon expertise.^25^

It is worth mentioning that great importance was given to the control of biases in the present study. The results were obtained from a selection of patients with the same skeletal malocclusion, whose ortho-surgical treatments were all performed by the same team of orthodontists and maxillofacial surgeons, with the same choice of surgery and fixation. The mandibular condyles behaved stably, and an adaptive process occurred in response to the mandibular advancement surgery performed.

SNA and SNB angles were also evaluated for anteroposterior skeletal discrepancies between maxilla and mandible, in order to control possible biases and characterize the sample. Effective control of the surgery was observed when analyzing the results obtained. When evaluating SNA-angle values, it was possible to conclude that the maxilla did not change during surgery. There was also a noticeable control in mandibular angle, ensuring good mandible positioning, and the SNB angle had an average change from 74° to 78°, showing control of the mandibular advancement procedure performed.

Mandibular condyle stability, one of the determining factors for a successful orthognathic surgery^25^ post-operative period, was supported by the results achieved, which showed no significant variation. This fact meets the recommendation of Park et al.^26^, who stated that condylar displacement within physiological capacity does not lead to morphological changes or condyle dysfunction.^26^

Furthermore, previous studies also confirm our findings. Draenert et al.^27^ and Barone et al.^22^ observed no significant changes in intercondylar distance and intercondylar angles in their 3D analysis. Lee and Park^4^ suggested that the fixation method and the surgical technique can influence the intercondylar distance. All cases from our series of cases followed the same surgical and fixation techniques.

This study is important for professionals working in the area, as it shows that the result obtained is beneficial. The presence of non-significant changes, obtained through angular and linear measurements, shows that the ortho-surgical procedure performed did not generate condylar structural and positional changes in patients who underwent mandibular advancement surgery, contributing to post-treatment stability. Furthermore, mandibular condyles on the right and left sides behaved similarly, suggesting stability and condylar adaptation after surgery.

Further studies should be performed with a greater number of cases and a larger post-operative follow-up period, to better understand the condylar changes.

## CONCLUSION

After evaluating the CBCT, it can be concluded that the spatial position of the mandibular condyle in the mandibular fossa remained unchanged after mandibular advancement surgery, contributing to post-surgical stability. The structure, position and dimensions of right and left mandibular condyles did not show major changes, suggesting physiological adaptation inherent in the surgical movement performed.
